# The Perspectives of Biomarkers in Predicting the Survival of the Renal Graft

**DOI:** 10.3389/fped.2022.869628

**Published:** 2022-06-03

**Authors:** Paul Luchian Aldea, Andreea Liana Rachisan, Bogdan Ioan Stanciu, Andrei Picos, Alina Monica Picos, Dan Ioan Delean, Ramona Stroescu, Magdalena Iuliana Starcea, Cristina Maria Borzan, Florin Ioan Elec

**Affiliations:** ^1^Department of Community Medicine, Discipline of Public Health and Management, Iuliu Haţieganu University of Medicine and Pharmacy, Cluj-Napoca, Romania; ^2^Department of Mother and Child, Discipline of Pediatrics II, Iuliu Haţieganu University of Medicine and Pharmacy, Cluj-Napoca, Romania; ^3^Department of Radiology, Iuliu Haţieganu University of Medicine and Pharmacy, Cluj-Napoca, Romania; ^4^Department of Prevention in Dental Medicine, Iuliu Haţieganu University of Medicine and Pharmacy, Cluj-Napoca, Romania; ^5^Department of Dental Prosthetics, Iuliu Haţieganu University of Medicine and Pharmacy, Cluj-Napoca, Romania; ^6^Department of Pediatrics, Victor Babeş University of Medicine and Pharmacy, Timisoara, Romania; ^7^Department of Pediatrics, University of Medicine & Pharmacy “Grigore T. Popa”, Iasi, Romania; ^8^Department of Surgical Sciences, Discipline of Urology, Iuliu Haţieganu University of Medicine and Pharmacy, Cluj-Napoca, Romania

**Keywords:** cystatin C, neutrophil gelatinase-related lipoprotein, kidney injury molecule 1, beta 2 microglobulin, biomarkers, graft function

## Abstract

Kidney transplantation (KT) is currently the elective approach for patients with end-stage renal disease. Although it is a safe choice for these patients, the early complications can lead to graft dysfunction. One of the most redoubtable complications is delayed graft function (DGF), having no specific treatment. The effects of DGF on the graft survival are large enough to justify the formulation of specific biological protocols. Therefore, discovering biomarkers of acute impairment in renal transplanted patients is required. Creatinine is a poor marker to establish the kidney injury. Estimated glomerular filtration rate together with creatinine is ready to approximately measure the kidney function. Different serum and urine proteins are being studied as possible predictive biomarkers for delayed graft function. This review will concentrate on recent and existing research which provide insight concerning the contribution of some molecules for the estimation and evaluation of graft function after kidney transplantation. Further studies examining various aspects of DGF after KT are urgently needed to address a hitherto less-known clinical question.

## Introduction

**Figure 1:**
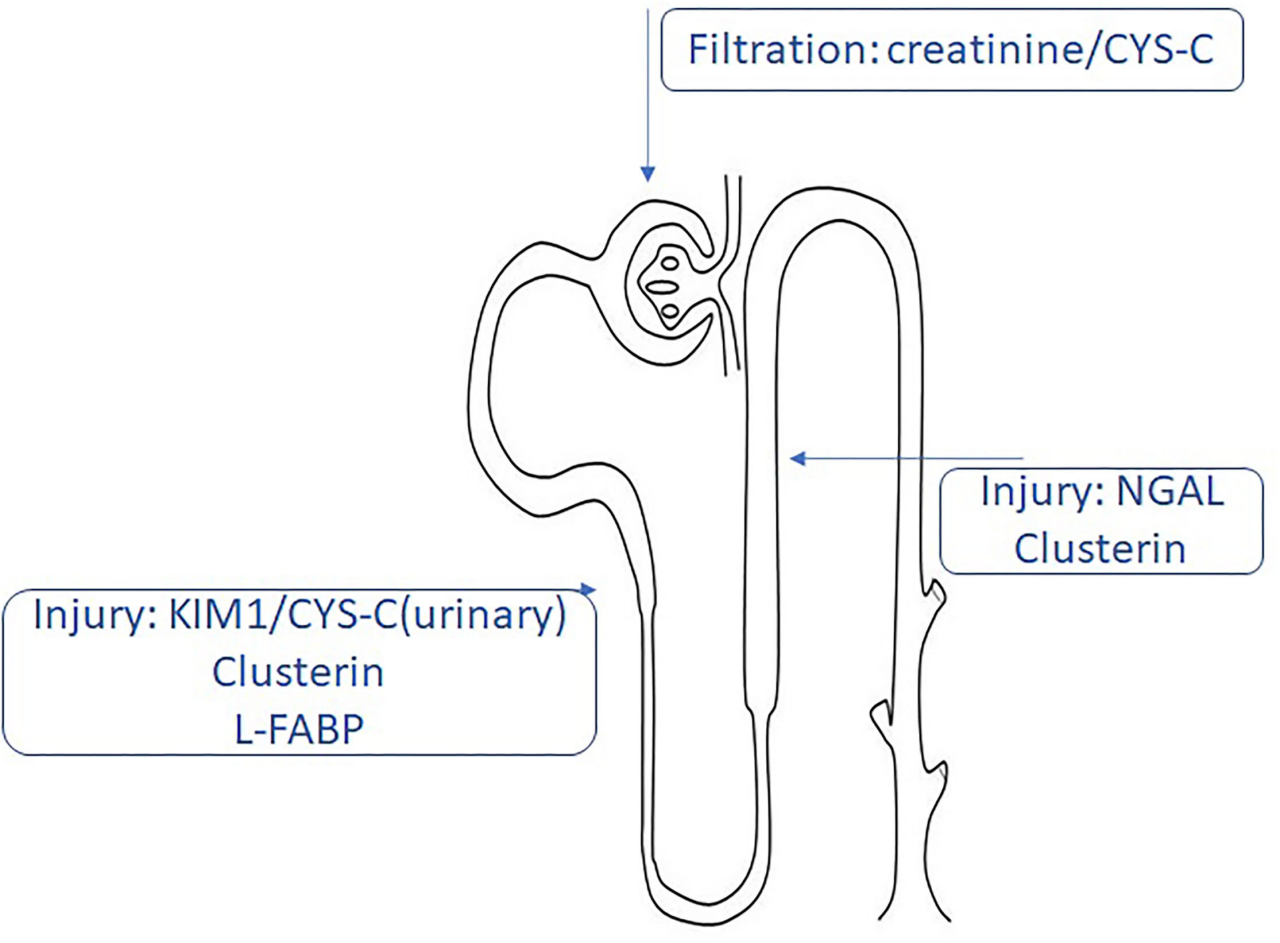
Traditional and new biomarkers that can indicate kidney damage.

Kidney transplantation (KT) is the elective approach in chronic kidney disease (CKD) stage V, and it provides a better quality of life compared to extrarenal epuration methods (e.g., hemodialysis) (1). The first year after transplantation is not absolved by complications. Although the surgical techniques are safe and the immunosuppressive protocols are standardized, patients with KT are unique and can develop complications from acute tubular necrosis to delayed graft function (DGF). Therefore, the development of long-term complications after KT is still seen (2). DGF is defined based on the creatinine levels and the need for dialysis after KT (3). Among all the definitions, the most used and accepted one is based on the need of minimum one dialysis during the first week after KT (4). DGF was associated with higher rejection rates and worse in the short-term and long-term results due to miscellaneous factors including donor-related factors (donation after brain death, cold ischemic time, shipping distance, donor age, body mass index, and others), recipient-related factors (preemptive or non-preemptive KT, previous KT, the presence of antibodies, ABO incompatibility, history of diabetes, recipient sex, and so on), and perioperative risk factors. DGF is usually associated with innate immune response because of complement activation and other molecular pathways activated during ischemic injury. The proposed mechanism suggests the release of inflammatory mediators *via* endothelial cells upregulating cell adhesion molecules (5). Currently, the evaluation of the renal graft is based on creatinine levels, the calculation of glomerular filtration rate (GFR), and the appearance of proteinuria. Being the *gold standard* assessment of the kidney function, creatinine and GFR are nonspecific markers, and the reliability is affected by several factors (6). Studies have focused on kidney injury molecules such as neutrophil gelatinase-related lipoprotein (NGAL), beta 2 microglobulin (β2MG), kidney injury molecule 1 (KIM1), and others, as the potential markers for the prognosis of graft durability (7). This paper will present different biomarkers for the evaluation of renal graft function and their potential role in the prediction of DGF, seeking to underline their limits and strengths (Table 1, Figure 1).

**Table 1:** Biomarkers in kidney transplantation.

**Biomarker**	**Abbreviations**	**Type of sample**
Creatinine	-	Serum/urine
Cystatin C	CYS-C	Serum/urine
Neutrophil gelatinase-related lipoprotein	NGAL	Serum/urine
Beta 2 microglobulin	B2MG	Serum/urine
Kidney injury molecule 1	KIM1	Urine
Uromodulin	UMOD	Serum
Clusterin	-	Serum/urine
Chitinase-3-like protein 1	YKL-40	Serum/urine
Liver-type fatty acid-binding protein	L-FABP	Urine

## Traditional Markers of Renal Function

### Creatinine

Creatinine is the first marker used to assess the kidney function and remains the most utilized test for the estimation of GFR. It is still considered to be the gold standard in the clinical practice, but it is not the most reliable due to many factors that contribute to its variability, including sex, musculature, medications, diet, etc. Several acute and chronic renal entities may exist without the modification of the creatinine baseline. The creatinine is not reflecting the tubular damage; thus, a preexisting lesion may be accompanied by a normal creatinine serum level (8). Although it is not the best predictor, creatinine serum level remains the marker used for the definition of acute kidney injury. The main cause may be the absence of primary validated markers of renal injury (9).

### Cystatin C

Cystatin C, also known as cystatin 3 (10), is a low molecular weight (13 kDa) protein containing 122 amino acid residues forming a single polypeptide sequence (11). Cystatin C is being a part of the cysteine protease inhibitors family (12), having the main activity the prevention of uncontrolled proteolysis and tissue damage (11). Cystatin C is thought to be generated by all human nucleated cells (11), being filtered by the glomerulus, and reabsorbed at the proximal tubular level, so it can be detected in the urine only in the context of renal injury (13). Plasma cystatin C is a marker of glomerular cell insult, having a role in impaired glomerular filtration rate (14). Many studies investigated urinary cystatin C in patients undergoing cardiac surgery, in the context of contrast administration, and they showed that it is an early diagnostic biomarker of acute kidney injury (AKI) in different settings, having elevated values 2 days before the AKI is installed. On the other hand, serum cystatin C cannot predict the complications in AKI (14). A meta-analysis published by Yong et al. (15) demonstrated that serum cystatin C can be a good diagnostic tool for the prediction of all-cause AKI (post-cardiac surgery and contrast-induced nephropathy), with an overall diagnostic sensitivity and specificity of 82 and 82%, respectively. In addition, Dos Santos Gomes et al. (16) evaluated kidney function markers in pregnancy, comparing 38 women with preeclampsia vs. 22 controlled pregnant women, showing that both urine and plasma cystatin C levels were significantly higher in the preeclampsia group compared to the control group, suggesting that cystatin C could be a reliable marker of kidney damage expressed by glomerular injury in preeclampsia. In addition, Vijay et al. (17) showed that plasmatic cystatin C level had the highest diagnostic reliability of AKI among children with liver cirrhosis, especially in those with decompensation or spontaneous bacterial peritonitis; it was also a reliant predictor of AKI in the pediatric population with liver cirrhosis, identifying AKI at an early stage. The same study showed that estimated glomerular filtration rate (eGFR) using serum cystatin C-based formulas was more predictable than that estimated by creatinine-based equations (17). Cystatin C has been proposed as a functional biomarker of glomerular filtration rate, with increased sensitivity in detecting early kidney dysfunction (within 24 h) compared to serum creatinine (1, 12). Therefore, it is a very useful biomarker in chronic kidney disease (CKD), and the GFR based on cystatin C is more specific (14). The utilization of cystatin C as a biomarker in eGFR calculations has several benefits (it may provide an augmented ability to predict the risk of adverse outcomes, less interindividual variability), but also the limitations (worse test–retest reliability, higher costs). A potential advantage of cystatin C is that being a large molecule, the blood levels may rise earlier than creatinine; therefore, it can be a better predictor for the cardiovascular disease risk (18). Furthermore, cystatin C is not affected by preanalytical factors, such as age, gender, race, diet, and body muscle mass (2, 19). Unfortunately, cystatin C levels may be modified in thyroid disease, malignancy, inflammation, diabetes, smoking, increased body mass index, or following corticosteroid therapy (20–22). Despite its known role in the assessment of renal function, cystatin C was reported as a potential marker of acute kidney injury (23, 24). It has an increase in the first 12 h with a peak value at 24 h, returning to baseline in the next hours (25, 26). The 2012 Kidney Disease Outcomes Quality Initiative Clinical Practice Guideline suggested using the serum cystatin C in patients with GFR between 45 and 59 ml/min/1.73 m^2^ (26, 27). Regarding kidney transplant patients, a meta-analysis by Pan et al. showed that although there is a difference in sensitivity and specificity for the diagnosis of acute kidney injury between cystatin C and creatinine (cystatin C having an increased sensitivity but a reduced specificity compared to creatinine), the two correlate very well with glomerular filtration rate post-transplantation. In addition, it has been shown that at glomerular filtration rate values ≤ 80 ml/min/1.73 m^2^, cystatin C has a better ability to detect renal function after kidney transplantation and better efficiency in terms of exclusion diagnosis. However, the differences between detection methods of the two parameters (cystatin c and creatinine) influenced considerably the heterogeneity within the results of this meta-analysis (28).

## Novel Biomarkers of Kidney Injury

### Neutrophil Gelatinase-Associated Lipocalin (NGAL)

Neutrophile gelatinase-associated lipocalin (NGAL), also known as siderocalin, lipocalin 2 oroncogene 24p (29), it is a 25 kDa protein associated with human neutrophil gelatinase being a part of the lipocalins family (30). NGAL exists in three forms: a 25-kDa monomer, a 45-kDa homodimer, and conjugated to gelatinase as a 135-kDa heterodimer (29). NGAL has a bacteriostatic role: it binds to bacterial iron siderophores, inhibiting the bacterial iron uptake. Besides its bacteriostatic effect, NGAL exerts an antiapoptotic effect and stimulates renal tubular cell proliferation, suggesting a potential protective effect in AKI. NGAL was found in many organs, such as kidney, lung, large intestine, uterus, prostate, salivary gland, trachea, and stomach (29). Its biodisponibility augments with age and levels are higher in women compared to men (30). NGAL is a urinary marker produced especially by neutrophils, loop of Henle, and collecting ducts, but can also be detected in the epithelium of the proximal convoluted tubule (due to megalin-mediated malabsorption of NGAL). The functional roles of NGAL in the kidney include iron-trafficking, tubular epithelial genesis, anti-inflammatory, and antiapoptotic (31). It is liberated from lysosomes, brush-border, and cytoplasm of proximal tubular epithelial cells, secondary to injury, being a specific lane for the progression of kidney disease (32, 33). NGAL has been the most widely investigated of the available AKI biomarkers (32). Scientific proofs showed that plasma and urine NGAL are present approximately 2 days before the AKI develops, therefore being an early diagnostic biomarker in kidney injury and a useful tool for the risk stratification in chronic kidney disease (CKD) (33). A study published by Zhang et al. (34) compared the serum NGAL and creatinine levels between 38 critically ill patients with AKI and 38 critically ill patients without AKI, showing remarkably higher levels of NGAL in the first group than in the control group, making NGAL an early AKI diagnosis marker (sensitivity 90.2%; specificity 89.5%). Moreover, urinary NGAL measured at the onset of AKI can precisely predict persistent AKI, new-start CKD, and CKD progression in patients with AKI; therefore, it is a valuable instrument for the better assessment of AKI risk stratification (35, 36). A systematic review and meta-analysis assessed the implication of NGAL in diabetic kidney disease (DKD), and accumulated evidence from observational and cohort studies demonstrated that urine NGAL could early differentiate patients with DKD from controls, but the diagnostic value of urine NGAL in DKD still needs to be further evaluated (37). The predictive value of NGAL is influenced by standard renal function, AKI severity, age, inflammatory conditions, preeclampsia, and cancer (32). Other factors that can interfere with NGAL values are age, sex (female), urinary infections, and impaired renal function (CKD) (31). False-positive levels of NGAL seem to be found in patients with septic shock (38). A recent study conducted by Soveri et al. (39) analyzed day-to-day intraindividual variation in some urine markers and showed that NGAL must change by 83.3% before being considered clinically significant in patients with CKD. The role of NGAL in renal obstruction remains unclear, but significant reduction in plasma and urinary NGAL levels in patients with acute ureteric colic undergoing surgical management or spontaneous stone passage suggests the potential role of NAGL as a marker of relief of renal obstruction due to ureteric stones (40). Multiple studies showed the use of NGAL in the diagnosis of DGF (Table 2). Hall et al. (41) noted that serum NGAL was ineffective to distinguish injury in DGF patients and those with normal graft function. Bataille et al. (42) studied the accuracy of NGAL in the prediction of DGF with a sensitivity of 93.3% and a specificity of 88.5%, being more predictive than the plasma creatinine. In a study including 59 patients with KT [Lee et al. (43)], the patients were divided into DGF patients and immediate graft function (IGF) group. The serum NGAL was higher at any time in DGF patients compared with IGF. Compared to creatinine which had an AUC of 0.65, NGAL had an AUC of 0.86, with a sensitivity of 78.6% and a specificity of 77.8%. Buemi et al. examined the predictive accuracy of urinary and plasma NGAL in transplant patients (deceased and living). The plasma NGAL levels can be more specific than the urinary NGAL for the prediction of DGF (44). A study by Cantaluppi et al. demonstrated that plasma NGAL levels are a useful early biomarker for the detection of DGF in the first 24 h post-kidney transplant. Although plasma NGAL levels are strongly influenced by inflammatory states or the existence of chronic kidney disease (this study confirming this observation – NGAL levels before transplantation were high, being comparable to those detected in the DGF group: 662.7 ± 97.2 ng/ml vs. 632 ± 84 ng/ml), this marker was useful in predicting functional recovery. However, a more rapid decrease in serum NGAL compared to creatinine was observed in the first days post-transplant. NGAL has also been shown to be a useful and superior marker to creatinine in monitoring nephrotoxicity of calcineurin inhibitors (tacrolimus) in the post-transplant period (45).

**Table 2:** Comparison of studies on serum NGAL for the diagnosis of DGF after kidney transplantation.

**Group/year**	**Study characteristics**	**Time of measurement after KT**	**Remarks**
Hall et al. (41)	78 KT 26 DGF	0-24-48h	NGAL was not different between KT with DGF and others
Bataille et al. (42)	41 KT 15 DGF	24h	NGAL level early and precisely predicted DGF after KT
Lee et al. (43)	59 KT 14 DGF	24h	NGAL is higher in DGF patients at any time after KT
Buemi et al. (44)	97 KT 20 DGF	6-24-48h	NGAL levels were notably lower in LDs than in DDs. No DGF was found among LD kidney recipients, but DGF was seen in 25% of patients in the DD group

^*^LD, living donors; DD, deceased donors.

### Beta 2 Microglobulin (β2MG)

Beta 2 microglobulin is a low molecular weight protein (11, 8 kDa) (46) consisting of 100 amino acid proteins. β2MG is produced by all cells expressing MHC-1 antigens, but lymphocytes and tumor cells are presumed to be major biosynthetic sites (47). During normal cell turnover, β2MG is released in blood, synovial, cerebrospinal, amniotic, and seminal fluid, as well as in aqueous humor, colostrum, and saliva (46). Synthesis is increased in pathologies with high cell turnover (e.g., infections, auto-immune diseases, or other hemato-oncological entities) (47). For example, serum β2MG levels are high in leukemia, lymphoma, and multiple myeloma, despite preserved renal function, but also in solid cancers, and it is associated with poor prognosis in most of them. Moreover, serum β2MG is elevated in systemic lupus erythematosus or Still disease, hemophagocytic lymphohistiocytosis, and Sjögren's syndrome (48). β2MG is a urinary biomarker filtered by the glomerulus and reabsorbed completely in the proximal tubular setting, so it can be detected in the urine only following epithelial cell injury (49, 50). Even though low levels of β2MG are found in urine and serum of normal subjects, these levels might increase in the context of kidney injury due to decreased reabsorbance by the damaged tubules (13). β2MG is a good marker to assess renal function in adults, with similar results such as creatinine-based estimating equations, but it might be vigorously associated with cardiovascular morbidity and mortality than creatinine (50). Urinary β2MG increases after the administration of cisplatin, cyclosporine, or gentamicin, but the pathway between increased urinary β2MG and the development of AKI remains uncertain (49). A total of eighty-nine children aged between 2 months and 14 years with acute pyelonephritis without history of urinary infections were evaluated to determine the urinary β2MG diagnostic accuracy in detection and prediction of renal injury and scar. The cutoff point for urinary β2MG for the prediction of positive DMSA (technetium Tc 99m dimercaptosuccinic acid) scan was 0.8 mg, but it was not enough sensitive (40.9%) and specific (84.1%) to be used as a diagnostic marker for the prognosis of renal injury (51). Recently, Puthiyottil et al. (52) showed that in adult patients who remained alive after AKI, the urinary β2MG/creatinine ratio at 2 weeks was higher compared to control group, and it is predictive of low estimated glomerular filtration rate at 1 year. Furthermore, urine level β2MG in resuscitated patients after cardiac arrest at admission and day 3 were independently associated with high risk of AKI, mortality, and poor neurological outcome in a study published by Beitland et al. (53). Serum β2MG level may be used as a prognostic biomarker of renal decline in patients with type 2 diabetes (54), whereas β2MG mRNA expression in cells of the urinary sediment is higher in patients with type 1 diabetes with diabetic kidney disease in comparison with healthy subjects, demonstrating a tubulointerstitial damage promoted by albumin (55).

### Kidney Injury Molecule 1 (KIM1)

Kidney injury molecule 1 is a transmembrane protein, which consists of two portions – an extracellular portion and a cytoplasmic one. The KIM1 gene can be found on chromosome 5p33.3 and contains 14 exons (56). Another names for KIM1 are T-cell immunoglobulin mucin receptor 1 (TIM1) or hepatitis A virus cellular receptor 1 (HAVCR1). This biomarker is not expressed only in the kidney, but also in the liver and spleen. Recent studies showed that KIM1 is expressed only in renal injury, so this biomarker can be used for early diagnosis of kidney damage (57). Under conditions that cause acute kidney injury (conditions such as ischemia, hypoxia, toxicity, tubular interstitial diseases, and polycystic kidney disease), urinary and renal KIM1 levels increase depending on the extent of the damage (56). Following the renal tubullar cell injury, the ectodomain of KIM1 is released and is excreted in the urine and blood, a process mediated by metalloproteinases. KIM1 levels are also correlated with the decline of the GFR and kidney injury (58). In acute renal tubular damage, KIM1 promotes cell phagocytosis and the repair of tubular cells, and it also inhibits the renal inflammatory response. On the other hand, the constant increase in KIM1 levels in CKD is not a protective factor, causing the development of renal fibrosis and tubular apoptosis, and can increase the inflammatory response (56). One study showed that KIM1 does not predict mortality in pediatric AKI, but it shows mild performance in the prognosis of renal replacement therapy (59). Urinary KIM1 levels are higher in children with stage 2–3 AKI compared to the control group, but in stage 1 AKI, this was true only in the first 12 h of admission (60). In the ancient articles, for example, Marcus et al. (61) showed that there is no correlation between urinary KIM1 levels and DGF (61). Zhang et al. demonstrated that expression of KIM1 is correlated with the degree of kidney damage. KIM1 can also be involved in regeneration processes and can be considered a useful marker of renal repair (62). In a study conducted on 140 renal transplanted patients, 37 of whom had DGF, Zhu et al. (63) demonstrated that urinary KIM1 levels among DGF patients were higher than among IGF (immediate graft function) patients at 0 h post-transplantation, as well as on the first day post-transplantation, indicating that recipients with increased urinary KIM1 levels after the first post-transplant day have a 23.5% higher risk of developing DGF and a 27.3% higher risk of long-term graft dysfunction (63). Yadav et al. (64) concluded that urinary KIM1 is higher in DGF patients compared with IGF patients at 6–12–18–24 and 48 h after transplantation, having the 100% specificity and 89.9% sensitivity to predict DGF in the first 18 h after KT. Tavernier et al. (65) in a large study with 244 kidney graft recipients determined the urinary KIM1 10 days after transplantation and found a significant correlation with time of cold ischemia and DGF and also the serum creatinine (Table 3).

**Table 3:** Comparison of studies on urinary KIM1 for the diagnosis of DGF after kidney transplantation.

**Group/year**	**Study characteristics**	**Time of measurement after KT**	**Remarks**
Zhu et al. (63)	140 KT 37 DGF	0-24h	Urinary KIM1 in the DGF group were higher than those in the immediate graft function (IGF) recipients immediately post-transplantation and in the first 24 h post-transplantation
Yadav et al. (64)	56KT 9 DGF	0-6-12-1/8-24-48h	Urinary KIM1 levels were notably high at 6, 12, 18, 24, and 48 h in patients with DGF versus IGF
Tavernier et al. (65)	244 KT	10 days	Urinary KIM1 was remarkably associated with cold ischemia time, *delayed graft function*, and plasma creatinine 10 days after transplantation

### Uromodulin (UMOD)

Uromodulin, also known as Tamm–Horsfall protein (THP), is exclusively produced by renal epithelial cells. Levels of UMOD in the urine and in the blood are the valuable biomarkers to assses the tubular mass and renal function (66, 67). Despite being the most common urinary protein, its function remains uncertain, but some studies suggest that this protein might have a role in salt transport, in the protection against urinary tract infection and kidney stones (by reducing the aggregation of calcium crystals). Uromodulin also plays the role in kidney insult (acute and chronic) and innate immunity (by binding immunoglobulins) (68). In humans, uromodulin is encoded by the UMOD gene, which is located on chromosome 16. Some mutations in UMOD can cause autosomal dominant tubulointerstitial kidney disease (ADTKD), leading to the acummulation of mutant uromodulin in the endoplasmic reticulum of tubular cells, causing decresed levels of urinary uromodulin and tubulointerstitial injury. Mutations in UMOD gene can be also associated with the autosomal dominant renal disorder medullary cystic kidney disease-2 (MCKD2). Furthermore, genome studies have identified common variants in UMOD that can be associated higher risk of CKD and cardiovascular disease. But further research needs to be done to understand these findings (68). Some studies showed that patients with CKD with interstitial fibrosis and tubular atrophy have lower levels of uromodulin. Therefore, UMOD may represent intact renal mass better than kidney function and can be used for the recognition of incipient of CKD (69). Urinary uromodulin was associated with rapid decline of eGFR, being an independent predictor of rapid kidney function loss (70). There are many questions that need to be aswered about the processes that sustain the production of uromodulin and its role in different diseases such as CKD, nephrolithiasis, and UTI. In KT recipients, there is described an association of low levels of uromodulin levels in accordance with graft failure (71). Kemmner et al. included a large cohort of 239 KT recipients, among them 64 experienced DGF. They determined serum uromodulin pre-KT and 24 h after KT. The serum uromodulin was not higher in DGF patients (72). Recent studies regarding uromodulin are controversial; therefore, it is hard to establish its potential role in predicting the graft survival.

### Clusterin

Clusterin, also named apolipoprotein J, is an omnipresent glycoprotein present in three isoforms, all of them differing in their functions. Discovered almost four decades ago in ram rete testis fluid with the ability to cause clustering of red blood cells – hence the name, this multifunctional protein as of today is still an enigma (73). In humans, clusterin is coded by a gene localized on the chromosome 8 (74). The translation of this gene results in three mRNA isoforms with different localizations: expressed in several tissues and present in the extracellular space and various body fluids. The nuclear isoform, a truncated form of clusterin, can promote apoptosis (75–77) whereas the mitochondrial isoform has the opposite effect by suppressing BAX-dependent release of cytochrome c into the cytoplasm, thus inhibiting apoptosis (78). The secreted form of clusterin acts as an extracellular chaperone that forms stable and soluble complexes with misfolded proteins, playing a key role in the extracellular proteostasis system by facilitating the clearance of misfolded proteins (79). Emerging data suggest that clusterin plays an important role in many different diseases. There are several studies that show its involvement in neuroprotection, cancer along with chemotherapy resistance, cardioprotection, addictive behavior development, and pain modulation. The involvement of clusterin in the pathophysiology of Alzheimer's disease is one of the most studied biological roles of this protein (80). Due to its molecular size, the urinary clusterin level is specific for kidney (81). Several studies compared clusterin with traditional markers such as blood urea nitrogen or serum creatinine (82). Clusterin also appears to be a rational indicator of tubulointerstitial renal lesions in patients with pediatrics with lupus nephritis and demonstrates the potentiality to predict ESRD (83). One study – regarding different biomarker levels in drug-induced kidney insult, suggests that clusterin levels can be consistent with the severity grades of proximal tubular injury (82). Moreover, clusterin appears to be an encouraging biomarker in the management of diabetic kidney disease as the urinary levels of clusterin are associated with the severity of diabetic nephropathy in patients with diabetes (84).

### YKL-40

YKL-40, also known as chitinase-3-like protein 1, is a glycoprotein encoded by the CHI3L1 gene located on chromosome 1. YKL-40 is expressed and secreted by different cell types with high cellular activity (85, 86). Expression of YKL-40 is also found to be high in embryonic and fetal tissues known to have rapid proliferation and marked differentiation, and in tissues undergoing morphogenetic changes (85). YKL-40 plays a major role in tissue damage, inflammation, tissue repair and remodeling responses (87, 88), protection against apoptosis, and stimulation of angiogenesis. Studies show that YKL-40 modulates renal repair mechanisms after ischemic kidney injury in mice and showed to be a useful marker of kidney damage in kidney transplantation in man (89). In patients with nephrotic syndrome, serum YKL-40 levels are associated with endothelial dysfunction and increased arterial stiffness and may predict proteinuria levels for these patients (90). In hemodialysis patients, YKL-40 levels significantly improved risk prediction for all-cause and cardiovascular mortality compared to other cytokines. thus better reflecting inflammatory activity (91). YKL-40 is a protein that can be measured in urine on the first day of clinically manifested AKI and combining with other biomarkers –such as NGAL – could refine AKI prognosis and better assess renal injury repair (92).

### Liver-Type Fatty Acid-Binding Protein (L-FABP)

Liver-type fatty acid-binding protein is a 14 kDa protein, which was at first identified in the hepatocytes and afterward was expressed in the human renal proximal tubule epithelium (93, 94). L-FABP seems to play a role in fatty acid homeostasis and expresses also an antioxidant effect (95, 96). In animal model studies, renal L-FABP showed a protection value for the tubulointerstitial damage and unilateral ureteral obstruction (94). Urinary proteins have a renal toxic effect, causing tubulointerstitial dysfunction and contributing to the progression of renal destruction. Albumin-bound free fatty acids may also contribute to tubulointerstitial damage. In the case of massive proteinuria, free fatty acids are overexpressed in the proximal convoluted tubule, inducing the generation of proinflammatory citokines, thus enhancing protein-induced tubulointerstitial injury (97). Urinary L-FABP can have a diagnostic and predictive value in patients with AKI, and it seems to be a helpful marker for the follow-up in CKD (98, 99). A study conducted on 92 patients with AKI compared to 62 patients without clinical evidence of AKI found notable modifications in urine L-FABP (94). Regarding diabetic nephropathy and renal injury, urinary L-FABP can be an early diagnostic parameter or prognostic marker of renal function. Numerous studies have demonstrated that L-FABP is a useful biomarker for both CKD and AKI. Furthermore, Nakamura et al. found that urinary L-FABP levels are elevated in patients with septic shock and are not correlated with the requirement for extrarenal epuration (94). In patiens with kidney transplant (KT), Yamamoto et al. (96) concluded that urinary L-FABP levels were higher in the immediate period after KT. Przybylowski et al. (100) indicated that urinary L-FABP could be a possible early marker for damaged kidney function in patients with KT. Nevertheless, Yang et al. (99) showed that urinary L-FABP could be useful for predicting poor graft outcome for ≤2 years; they showed that 0-h urinary L-FABP level was independently associated with DGF in patients with KT after 2 years. Their data indicate that urinary L-FABP might be useful for predicting adverse long-term graft outcomes.

### Donor-Derived Cell-Free DNA and Its Role in DGF

Donor-derived cell-free DNA (ddcf-DNA) is typically encountered in the body fluids of post-transplant individuals and refers to cell-free DNA that arises after apoptosis or necrosis of the allograft tissue. Therefore, ddcf-DNA can be used as a prospective biomarker to evaluate the status of donor tissues. Research findings indicate that levels above 1% ddcf-DNA in recipient plasma are an indicator of acute rejection risk in transplanted patients. There is no significant difference in early ddcf-DNA levels between patients with DGF and those without DGF. When compared to non-DGF subjects, patients with DGF have a 1.5-fold higher risk of renal allograft rejection. As such, earlier diagnosis of DGF and acute rejection is of critical importance for accurate and prompt clinical intervention. In patients with DGF and acute rejection, ddcf-DNA levels have been shown to drop and follow a comparable pattern in the early postoperative stages. However, when plasma ddcf-DNA levels in patients with DGF remain >1%, it might indicate acute rejection in renal transplanted patients. Most previously available studies support elevated ddcf-DNA levels in acute rejection. However, many other causes of high serum ddcf-DNA levels also exist, among them infection and acute tubular necrosis. They are both frequent occurrences in the renal transplanted population. In conclusion, ddcf-DNA acts as a marker of allograft injury, but it is not specific to any form of rejection. Increased levels could occur in allograft-limited conditions, including rejection, but can also be raised in systemic conditions, such as malignancy and infection (101, 102).

## Conclusions

These potential DFG biomarkers need supplementary validation and require more understanding. Although the roles of described molecules have been established as the markers of renal injury, there is limited application to translate benchwork to clinical use. We consider that there is no ideal renal injury biomarker, and only the combination of a panel containing different biomarkers can elucidate the DGF mechanism and can predict earlier this event to maximize the therapeutical strategies.

## Author Contributions

Conceptualization: PA, AR, BS, MS, RS, and FE. Validation: AR, CB, and AMP. Writing—original draft preparation: PA, AR, and AP. Writing, reviewing, and editing: AR, FE, MS, RS, and CB. Visualization: FE, CB, and AP. Supervision: FE, AR, CB, and DD. All authors have read and agreed to the published version of the manuscript.

## Funding

This work was supported by the University of Medicine & Pharmacy Iuliu Hatieganu Cluj-Napoca, Romania, project number 35150/17.12.2021-*Imunological and biological predictive factors for the renal graft survival – a pilot study*.

## Conflict of Interest

The authors declare that the research was conducted in the absence of any commercial or financial relationships that could be construed as a potential conflict of interest.

## Publisher's Note

All claims expressed in this article are solely those of the authors and do not necessarily represent those of their affiliated organizations, or those of the publisher, the editors and the reviewers. Any product that may be evaluated in this article, or claim that may be made by its manufacturer, is not guaranteed or endorsed by the publisher.
